# Supplementary material for: FacZ is a GpsB-interacting protein that prevents aberrant division-site placement in Staphylococcus aureus
Source: Nat Microbiol. 2024 Mar 5;9(3):801–13. doi: 10.1038/s41564-024-01607-y (PMC10914604; doi:10.1038/s41564-024-01607-y)
Supplement: Supplementary file 1 — Supplementary Tables 8–11, legends for Supplementary Tables 1–7 and References. [file 41564_2024_1607_MOESM1_ESM.pdf]

nature microbiology

Article https://doi.org/10.1038/s41564-024-01607-y

FacZ is a GpsB-interacting protein that prevents aberrant division-site placement in *Staphylococcus aureus*

In the format provided by the authors and unedited

Supporting information for:

Title: **FacZ is a GpsB-interacting protein that prevents aberrant division-site placement in *Staphylococcus aureus***

Authors: Thomas M. Bartlett[1], Tyler A. Sisley[1], Aaron Mychack[1], Suzanne Walker[1], Richard W. Baker[2,3], David Z. Rudner[1*], Thomas G. Bernhardt[1,4*]

Affiliations:

[1]Department of Microbiology, Blavatnik Institute, Harvard Medical School, Boston, Massachusetts, USA

[2]Department of Biochemistry & Biophysics, School of Medicine, University of North Carolina at Chapel Hill, Chapel Hill, NC, USA

[3]Lineberger Comprehensive Cancer Center, University of North Carolina at Chapel Hill, Chapel Hill, NC, USA

[4]Howard Hughes Medical Institute, Harvard Medical School, Boston, Massachusetts, USA

***To whom correspondence should be addressed:**

Thomas G. Bernhardt, Ph.D.
Harvard Medical School
Department of Microbiology
Boston, Massachusetts 02115
e-mail: thomas_bernhardt@hms.harvard.edu

David Z. Rudner
Harvard Medical School
Department of Microbiology
Boston, Massachusetts 02115, USA
e-mail: david_rudner@hms.harvard.edu

Supplemental Table Legends

Table S1. Tn-seq meta-analysis.

Table summarizes the relevant metrics from Tn-Seq analysis of the initial transposon library, END and CSD enrichments, and the ungated control sort. The numbers 2 and 3 refer to the sorting round (e.g., CSD 2 has been subjected to two rounds of sorting increased light scattering). The total number of unique TA sites and the percentage of total TA sites in the genome with transposon insertions are given, as well as median and mean number of insertions per gene in the library following enrichments. The number of genes in each library hit at least once, twice, or five times is shown at right, along with the percent of annotated genes bearing at least that many hits. Also, the number of genes meeting or exceeding these thresholds is compared to the number of mutants in the NTML ordered transposon library[2], which is commonly used in *S. aureus* research.

Table S2. Tn-seq data for the END and CSD enrichments.

Tn-seq results for the second and third rounds of sorting of the CSD and END enrichments. Relative enrichments compare the results of a given round of either CSD or END sorting to the ungated control. P-values are from Mann-Whitney U test. Genes are sorted by SAOUHSC locus number.

Table S3. Relative enrichment of transposon insertions in each gene following END and CSD enrichments versus the ungated control.

Tn-seq data showing the relative enrichments of the END and CSD sorts compared to an ungated control. Genes are sorted by SAOUHSC locus number.

Table S4. PCA-adjusted Tn-Seq results.

Following 2D-PCA rotation of the CSD and END sorted data, each gene's enrichment was assigned a new (X,Y) location, with X representing a genes relative enrichment along PC2, and Y representing the relative enrichment along PC1. These data were used to plot the PCA panel in **Fig. S1**. Genes are sorted by SAOUHSC locus number.

Table S5. PCA-adjusted hit list.

The top 50 hits from the PCA analysis based on maximum relative enrichment along PC1. "Δ" indicates the change in PC1 or PC2 relative enrichment between rounds of sorting. "Mean fold increase" and "% increasing in enrichment" are metrics to assess whether hits identified by PCA in sort 2 increase in PC1 enrichment from additional rounds of CSD and END sorting. In general, hits identified by PC1 enrichment enrich further from additional sorts, where those from PC2 do not, consistent with PC1-enrichment serving as a proxy for mutual CSD and END enrichment. Genes are displayed by decreasing relative enrichment along PC1.

Table S6. Δ*facZ* synthetic lethal Tn-seq hits

Tn-seq analysis of transposon libraries constructed in WT *S. aureus* [aTB015] versus one constructed in a Δ*facZ* [aTB259] strain. P-values are from Mann-Whitney U test. Genes are sorted by increasing Δ*facZ:WT* relative enrichment ratio.

Table S7. Annotated Δ*facZ* synthetic lethal Tn-seq hits

Tn-seq analysis of transposon libraries constructed in WT *S. aureus* [aTB015] versus one constructed in a Δ*facZ* [aTB259] strain. P-values are from Mann-Whitney U test. Genes are sorted by increasing Δ*facZ:WT* relative enrichment ratio. Only the top 50-most de-enriched loci from the Δ*facZ* synthetic lethal analysis are displayed; see **Table S6** for a complete list of unannotated loci. Loci were identified with gene names and brief descriptions from AureoWiki. Genes previously described as having a role in cell wall biosynthesis, morphogenesis, cell division, and cell separation are highlighted in yellow.

Table S8: ***S. aureus*** **strains used in this study**

***S. aureus* strain**	**Background**	**Relevant genotype**	**Source**	**Construction notes**
**aTB001**	USA300	WT	Pang, T. et al.[3]	-
**aTB003**	HG003	WT	Pang, T. et al.[3]	-
**aTB004**	RN4220	WT	Pang, T. et al.[3]	-
**aTB015**	RN4220	*ΔattB(f11)::Orf5, pTM378*	Wang, H. et al.[4]	-
**aTB016**	RN4220	*ΔattB(f11)::Orf5, pTM381*	Wang, H. et al.[4]	-
**aTB033**	RN4220	pTP044	Pang, T. et al.[3]	-
**aTB111**	USA300	*Tn5::USA300_0932 (SAOUHSC_00965)*	Fey, P. et al.[2]	-
**aTB112**	USA300	*Tn5::USA300_1792 (SAOUHSC_01975)*	Fey, P. et al.[2]	-
**aTB113**	USA300	*Tn5::USA300_2094 (SAOUHSC_02383)*	Fey, P. et al.[2]	-
**aTB209**	HG003	*Δspa::kan*	Pang, T. et al.[3]	-
**aTB219**	HG004	*pLOW-ftsZ-GFP*	This study	pLOW-ftsZ-GFP transduced into aTB003
**aTB243**	RN4220	*ΔfacZ::specR*	This study	Homologous recombination of pMR91-Δ*facZ::specR* into aTB004
**aTB251**	HG003	*ΔfacZ::specR*	This study	Φ85 lysate of aTB243 was used to transduce Δ*facZ::specR* into aTB003
**aTB259**	RN4220	*ΔfacZ::specR ΔattB(f11)::Orf5, pTM378*	This study	Φ85 lysate of aTB243 was used to transduce Δ*facZ::specR* into aTB015
**aTB261**	RN4220	*ΔfacZ::specR ΔattB(f11)::Orf5, pTM381*	This study	Φ85 lysate of aTB243 was used to transduce Δ*facZ::specR* into aTB016
**aTB263**	RN4220	*pGL485*	This study	pGL485 was transformed into aTB004
**aTB287**	HG003	*ΔsagB (lytD)*	This study	Provided by Walker lab
**aTB315**	RN4220	*pGL485 pLOW-facZ*	This study	*pLOW-facZ* was transformed into aTB263
**aTB316**	RN4220	*pGL485 pLOW-facZ-mCherry*	This study	*pLOW-facZ-mCherry* was transformed into aTB263
**aTB317**	RN4220	*pGL485 pLOW-facZ-6xHis*	This study	*pLOW-facZ-6xHis* was transformed into aTB263
**aTB339**	RN4220	*pTP044 pTP63d-facZ*	This study	*pTP63d-facZ* was transformed into aTB044
**aTB341**	HG003	*pTP63d-facZ*	This study	Φ85 lysate of aTB339 was used to transduce *pTP63-facZ* into aTB003
**aTB347**	HG003	*pLOW-facZ*	This study	Φ85 lysate of aTB315 was used to transduce *pLOW-facZ* into aTB003
**aTB348**	HG003	*pLOW-facZ-mCherry*	This study	Φ85 lysate of aTB316 was used to transduce *pTP63-facZ-mCherry* into aTB003
**aTB349**	HG003	*pLOW-facZ-6xHis*	This study	Φ85 lysate of aTB317 was used to transduce *pTP63-6xHis* into aTB003
**aTB356**	HG003	*ΔfacZ::specR pLOW-facZ*	This study	Φ85 lysate of aTB243 was used to transduce Δ*facZ::specR* into aTB347
**aTB358**	HG003	*ΔfacZ::specR pLOW-facZ-mCherry*	This study	Φ85 lysate of aTB243 was used to transduce Δ*facZ::specR* into aTB348
**aTB372**	HG003	*ΔfacZ::specR pTP63d-facZ*	This study	Φ85 lysate of aTB243 was used to transduce Δ*facZ::specR* into aTB341
**aTB374**	RN4220	*pTP044 pTP63d-facZ-mCherry*	This study	*pTP63d-facZ-mCherry* was transformed into aTB033
**aTB376**	RN4220	*pTP044 pTP63-ftsZ-gfp*	This study	*pTP63-ftsZ-gfp* was transformed into aTB033
**aTB378**	HG003	*ΔezrA pTP63b-ezrA ΔfacZ::specR*	This study	Φ85 lysate of aTB243 was used to transduce Δ*facZ::specR* into aTB481
**aTB390**	HG003	*ΔfacZ::specR pTP63d-facZ pLOW-FtsZ-GFP*	This study	Φ85 lysate of *RN4220 pLOW-ftsZ-GFP* was transduced into aTP372
**aTB391**	HG003	*ΔezrA pTP63d-ezrA pLOW-FtsZ-GFP*	This study	Φ85 lysate of *RN4220 pLOW-ftsZ-GFP* was transduced into aTP481
**aTB392**	HG003	*pTP63d-facZ-mCherry*	This study	Φ85 lysate of aTB374 used to transduce *pTP63-facZ-mCherry* into aTB003

**aTB394**	HG003	*pTP63d-ftsZ-GFP*	This study	Φ85 lysate of aTB376 used to transduce *pTP63-ftsZ-GFP* into aTB003
**aTB411**	HG003	*Δspa::kan pLOW-01855-6xHis*	This study	Transformed *pLOW-01855-6xHis* from RN4220 into aTB209
**aTB453**	HG003	*ΔfacZ::specR gpsB (T)6→5 (truncation 187)*	This study	Spontaneous suppressor of aTB251 sensitivity to PC190723
**aTB476**	HG003	*ΔfacZ::specR gpsB(Y26*)*	This study	Spontaneous suppressor of aTB251 sensitivity to PC190723
**aTB478**	HG003	*ΔfacZ::specR gpsB (T)6-5 (trunc. 128)*	This study	Spontaneous suppressor of aTB251 sensitivity to PC190723
**aTB492**	HG003	*gpsB::Tn5*	This study	Φ85 lysate of NTML strain *gpsB::Tn5(erm)* transduced into aTB003
**aTB497**	HG003	*gpsB::Tn5 ΔfacZ::specR*	This study	Φ85 lysate of aTB243 was used to transduce *ΔfacZ::specR* into aTB492
**aTB513**	HG003	*Δspa::kan pLOW-01855 pTP63-gpsB-FLAG*	This study	Transformed *pLOW-01855* from RN4220 into aTB209
**aTB514**	HG003	*Δspa::kan pLOW-01855-6xHis pTP63-gpsB-FLAG*	This study	Transformed *pLOW-01855-6xHis* from RN4220 into aTB209
**aTB515**	HG003	*pTP63-gpsB-mNeon*	This study	Φ85 lysate of AGS063 was used to transduce *pTP63-gpsB-mNeon* into aTB003
**aTB517**	HG003	*pTP63-gpsB-mNeon pLOW-01855-mCherry*	This study	Φ85 lysate of aTB316 was used to transduce *pLOW-facZ-mCherry* into aTB515
**aTB519**	HG003	*pTP63-gpsB-mNeon ΔfacZ::specR*	This study	Φ85 lysate of aTB243 was used to transduce *ΔfacZ::specR* into aTB515
**aTB521**	HG003	*pKK30_RFP*	This study	Φ85 lysate of RN4220 *pKK30_RFP* was used to transduce pKK30_RFP into aTB003
**aTB525**	HG003	*ΔgpsB::Kan*	This study	Φ85 lysate of TAS201 was used to transduce *ΔgpsB::Kan* into aTB003
**aTB527**	HG003	*ΔfacZ::specR pKK30_RFP*	This study	Φ85 lysate of aTB521 was used to transduce pKK30_RFP into aTB251
**aTB529**	HG003	*ΔgpsB::kanR pKK30_RFP*	This study	Φ85 lysate of aTB521 was used to transduce pKK30_RFP into TAS201
**aTB540**	HG003	*ΔfacZ::specR pTP63-facZ ΔgpsB::kanR*	This study	Φ85 lysate of TAS201 was used to transduce *ΔgpsB::kanR* into aTB372
**aTB542**	HG003	*ΔfacZ::specR ΔgpsB::kanR pKK30-RFP*	This study	Φ85 lysate of aTB243 was used to transduce *ΔfacZ::specR* into aTB529
**aTB549**	HG003	*ΔfacZ::specR pTP63-facZ pKK30-RFP*	This study	Φ85 lysate of aTB521 was used to transduce *pKK30_RFP* into aTB372
**aTB565**	RN4220	*pLOW-facZ(3R-3D)-6xHis ΔfacZ*	This study	Plasmid transformed into aTB243
**aTB568**	RN4220	*pLOW-facZ(R135D)-6xHis ΔfacZ*	This study	Plasmid transformed into aTB243
**aTB570**	RN4220	*pLOW-facZ(R138D)-6xHis ΔfacZ*	This study	Plasmid transformed into aTB243
**aTB572**	RN4220	*pLOW-facZ(R139D)-6xHis ΔfacZ*	This study	Plasmid transformed into aTB243
**aTB574**	RN4220	*pLOW-facZ(R160D)-6xHis ΔfacZ*	This study	Plasmid transformed into aTB243
**aTB632**	HG003	*pTP63-gpsB-FLAG*	This study	Provided by Walker lab
**aTB643**	RN4220	*pLOW-ftsW-GFP*	This study	*pLOW-ftsW-GFP* transformed into aTB004
**aTB645**	RN4220	*pLOW-GFP-pbpA*	This study	*pLOW-GFP-pbpA* transformed into aTB004
**aTB649**	HG003	*pTP63-gpsB-FLAG ΔfacZ::specR*	This study	Φ85 lysate of aTB243 was used to transduce *ΔfacZ::specR* into aTB632
**aTB651**	HG003	*pLOW-facZ(3R-3D)-6xHis*	This study	Φ85 lysate of aTB565 transduced into aTB251
**aTB663**	HG003	*gpsB::Tn5 ΔezrA::kanR pTP63-ezrA*	This study	Φ85 lysate of NTML strain *gpsB::Tn5(erm)* transduced into aTB264
**aTB665**	HG003	*pLOW-GFP-pbpA*	This study	Φ85 lysate of aTB645 was used to transduce *pLOW-GFP-pbpA* into aTB251
**aTB666**	HG003	*pLOW-ftsW-GFP*	This study	Φ85 lysate of aTB643 was used to transduce *pLOW-ftsW-GFP* into aTB251
**aTB673**	HG003	*ΔfacZ pLOW-GFP-pbpA*	This study	*pLOW-GFP-pbpA* purified from aTB645 and transformed into aTB251
**aTB675**	HG003	*ΔfacZ pLOW-ftsW-GFP*	This study	*pLOW-ftsW-GFP* purified from aTB643 and transformed into aTB251
**aTB679**	HG003	gpsB::Tn5 ΔezrA::kanR pTP63-ezrA ΔfacZ	This study	Φ85 lysate of aTB243 was used to transduce *ΔfacZ::specR* into aTB663
**aTP071**	RN4220	pTP10 integrant	This study	pTP10 transformed and integrated into RN4220; cloning intermediate for Δ*atl*

**aTP103**	HG003	Δ*atl*	This study	Φ85 lysate of aTP071 was used to transduce Δ*atl::kanR* into HG003
**aTP436**	RN4220	pTP044 pTP63-ezrA	This study	pTP63-ezrA transformed into RN4220 pTP044
**aTP455**	RN4220	pTP89 integrant	This study	pTP10 transformed and integrated into RN4220; cloning intermediate for Δ*ezrA*
**aTP481**	HG003	Δ*ezrA pTP63-ezrA*	This study	Unpublished (Rudner collection)
**TAS201**	RN4220	Δ*gpsB::Kan*	This study	Provided by Walker lab
**TAS079**	RN4220	*pLOW-gpsB-FLAG*	This study	Provided by Walker lab
**AGS063**	RN4220	*pTP63-gpsB-mNeon*	This study	Provided by Walker lab
**SB100**	RN4220	*pKK30-RFP*	This study	Provided by Walker lab

All *S. aureus* strains used in the course of this study are noted in this table. See Methods section for a description of how strains were made.

Table S9: *B. subtilis* strains used in this study

***B. subtilis* strain**	**Relevant Genotype**	**Source**	**Construction Notes**
bDR11	*WT*	Youngman, P. et al.[5]	-
bDR2229	*amyE::*P_{spac}*-ftsZ-gfp*	Ben-Yehuda, S. & Losick, R.[6]	-
bDR2637	*sacA::Pveg-mCherry(phleo)*	Roney, I. & Rudner, D.[7]	-
bDR2660	*sacA::Pveg-BFP(phleo)*	Roney, I. & Rudner, D.[7]	-
bDR2789	*sacA::Pveg-GFP(phleo)*	Roney, I. & Rudner, D.[7]	-
bTB013	*ΔfacZ::ermR*	This study	Marker crossed from deletion library into bDR11
bTB018	*amyE::Pspac-ftsZ-gfp ΔfacZ::ermR*	This study	Marker crossed from deletion library into bDR2229
bTB039	*sacA::Pveg-GFP(phleo) ΔfacZ::erm*	This study	Marker crossed from deletion library into bDR2789
bTB040	*sacA::Pveg-BFP(phleo) ΔfacZ::erm*	This study	Marker crossed from deletion library into bDR2660
bTB041	*sacA::Pveg-BFP(phleo) ΔfacZ::erm ΔgpsB::spec*	This study	Marker crossed from deletion library into bTB040
bTB044	*sacA::Pveg-GFP(phleo) ΔgpsB::spec*	This study	Marker crossed from deletion library into bDR2789

All *B. subtilis* strains used in the course of this study are noted in this table. See Methods section for a description of how strains were made.

Table S10: Plasmids used in this study

Plasmid	Relevant genotype & description	Marker	Source	Cut Sites/ ITA	Oligos
pTM378	ts origin, HMAR1 C9 transposase	Kan	Wang, H. et al.[4]	-	-
pTM381	ts origin, HMAR1 C9 transposase (truncated)	Kan	Wang, H. et al.[4]	-	-
pTP044	L54a integrase-bearing plasmid	Tet	Pang, T. et al.[3]	-	-
pLOW-ftsZ-GFP	*ftsZ-gfp* under P_{spac} promoter	Erm	Liew, A. et al.[8]	-	-
pLOW-GFP-ftsW	*gfp-ftsW* under P_{spac} promoter	Erm	This study	SalI/BamHI	oTB689-692
pLOW-pbp1-GFP	*pbp1-gfp* under P_{spac} promoter	Erm	This study	SalI/BamHI	oTB699-702
pGL485	High-copy plasmid constitutively expressing LacI	Cm	Liew, A. et al.[8]	-	-
pKK30	dsRed, constitutively expressed red fluorescence	Tmp	Rodriguez, M. et al. [9]	-	-
pLOW-facZ	*facZ* under P_{spac} promoter	Erm	Synthesized	-	-
pLOW-facZ-mCherry	*facZ-mCherry* under P_{spac} promoter	Erm	Synthesized	-	-
pLOW-facZ-6xHis	*facZ-6xHis* under P_{spac} promoter	Erm	Synthesized	-	-
pLOW-facZ(R135D)-6xHis	*facZ(R135D)-6xHis* under P_{spac} promoter	Erm	Synthesized	-	-
pLOW-facZ(R138D)-6xHis	*facZ(R138D)-6xHis* under P_{spac} promoter	Erm	Synthesized	-	-
pLOW-facZ(R139D)-6xHis	*facZ(R139D)-6xHis* under P_{spac} promoter	Erm	Synthesized	-	-
pLOW-facZ(R160D)-6xHis	*facZ(R160D)-6xHis* under P_{spac} promoter	Erm	Synthesized	-	-
pLOW-facZ(3R-3D)-6xHis	*facZ(R135D, R138D, R160D)-6xHis* under P_{spac} promoter	Erm	Synthesized	-	-
pLOW-gpsB-GFP	*gpsB-mNeon* under P_{spac} promoter	Erm	Walker lab	-	-
pTP63-lacZ	*lacZ* under P_{tet} promoter	Cm	Pang, T. et al.[3]	-	-
pTP63-ezrA	*ezrA* under P_{tet} promoter	Cm	Rudner lab, unpublished	-	-
pTP63-facZ	*facZ* under P_{tet} promoter	Cm	This study	KpnI/EcoRI	oTB561, 562
pTP63-facZ-mCherry	*facZ-mCherry* under P_{tet} promoter	Cm	This study	KpnI/EcoRI	oTB563, 564
pTP63-gpsB-mNeon	*gpsB-mNeon-FLAG* under P_{tet} promoter	Cm	This study	ITA	AGS059-064
pTP63-ftsZ-GFP	*ftsZ-GFP* under P_{tet} promoter	Cm	This study	KpnI/EcoRI	oTB566, 568
pMR91-ΔfacZ::specR	1.5 kb flanking sequence of *facZ* interrupted with *specR* marker, constitutive mScarlett, ts origin	Erm, Spec	This study	ITA	oTB490, 491, 492, 493
pLOW-gpsB-FLAG	*gpsB-FLAG* under P_{spac} promoter	Erm	This study	SalI/BamHI	oTS079, 80
pSUMO-FacZ 3x(127-146)	Purification plasmid for His-SUMO-tagged $FacZ_{(127\text{-}146)}$	Kan	This study	ITA	oRWB137, oRWB138

pSUMO-GpsB (1-75)	Purification plasmid for His-SUMO-tagged GpsB $FacZ_{(1-75)}$	Kan	This study	ITA	oRWB123, oRWB124

All plasmids produced in the course of this study were made by isothermal assembly (ITA) or restriction digest and ligation using pairs of restriction enzymes, as noted in this table. Plasmids were isolated from and are stored in either DH5α or BL21(DE3) *E. coli* strains, and are available upon request.

**Table S11: Oligos used in this study** **Oligo**	**Name**	**Sequence**	**Purpose**
**oTB045**	SpecR Seq F3	TGATTCCACGGTACCATTTCTTGC	Outward-facing sequencing primer for downstream homology arm of Δ*facZ::specR*
**oTB065**	SpecR Seq R4	AGTGCTCCCTGatGTCgacc	Outward-facing sequencing primer for upstream homology arm of Δ*facZ::specR*
**oTB169**	pMR091 MCS seq F	GACTTTACGAAACACGGAAACCG	Inward-facing sequencing primer for upstream homology arm of Δ*facZ::specR*
**oTB170**	pMR091 MCS seq R	atcagttcattgctcacgatatgtg	Inward-facing sequencing primer for downstream homology arm of Δ*facZ::specR*
**oTB192**	EzrA cPCR F	CATCTTCAATAAGGCTTGCTGC	Colony PCR for *ezrA* deletion
**oTB193**	EzrA cPCR R	CATCAGTCCAATTTGACAGAGTGC	Colony PCR for *ezrA* deletion
**oTB410**	pTP63_cPCR_F	CTCATTAAGCAGCTCTAATGCGC	Colony PCR for *pTP63* insert
**oTB411**	pTP63_cPCR_R	CCAGCGTTTCTGGGTGAGC	Colony PCR for *pTP63* insert
**oTB416**	del_Atl_cPCR_F1	GGCGAAGTCGGCAAATACTTCG	Colony PCR for *atl* deletion
**oTB417**	del_Atl_cPCR_R1	CGACGCATATCGTTGTAACACG	Colony PCR for *atl* deletion

**oTB490**	pMR091 01855 UpF	CGCTCGCGTATCGGTGATGGATCCCGATCAATTAGAGCAACTCGGTTATGTTTCG	Generate upstream flanking region of *facZ* for ITA
**oTB491**	pMR091 01855 UpR	cccggaaaaagagttgactaaatcaaTAAAAACGCCTCCTAATTAACATGTAATAATGTC	Generate upstream flanking region of *facZ* for ITA
**oTB492**	pMR091 01855 DnF	ccggtctatgttcatttagtcctccactaTTAATAATTAAACAAATGCACTTAAATGAGGTTGTTAC	Generate downstream flanking region of *facZ* for ITA
**oTB493**	pMR091 01855 DnR	gctctatataaaatatactcaaaatattatCCATGGtatGAATTCGCATTTGTTGTTTTTGAATTCTTATTACTAGTTTG	Generate downstream flanking region of *facZ* for ITA
**oTB494**	01855 up out F	GATGATGTTTGTGCATTTATGGTTAATGAAGG	Δ*facZ* sequencing primer
**oTB495**	01855 up in F	GATAATGCTGTTATTTTATTTATGGGTGCAGG	Δ*facZ* sequencing primer
**oTB496**	01855 dn in R	GTTGCTGCGTCATTTGTATATCCTCC	Δ*facZ* sequencing primer
**oTB497**	01855 dn out R	CATAGCTTACTGCTATGATTGATTATTCAACG	Δ*facZ* sequencing primer
**oTB498**	Spec Out Seq R	CAATAAACCCTTGCATAGGGATAACTTCG	Δ*facZ* sequencing primer
**oTB499**	Spec Out Seq F	CGTTACGTTATTAGTTATAGTTATTATAACATGTATTCACG	Δ*facZ* sequencing primer
**oTB500**	Spec Out Seq R2	GCAGTTCGTAGTTATCTTGGAGAGAATATTGAATG	Δ*facZ* sequencing primer
**oTB501**	Spec Out Seq F2	GTGTAAACCTATTCATTGTTTTAAAAATATCTCTTGCC	Δ*facZ* sequencing primer
**oTB513**	01855 outside f	GTTGATTTTATCAAACAACAAAGAGAACCGG	Colony PCR for *facZ* deletion
**oTB514**	01855 outside r	GGTAAGCACCTGAATGCCTACC	Colony PCR for *facZ* deletion
**oTB538**	pLOW MCS seq f1	GACTTTATCTACAAGGTGTGGC	Colony PCR *for pLOW* insert
**oTB539**	pLOW MCS seq f2	atcctctagagtcaattgtgagcgc	Colony PCR for *pLOW* insert
**oTB535**	PolyG-1st-1 primer	GTGACTGGAGTTCAGACGTGTGCTCTTCCGATCTGGGGGGGGGGGGGGGG	Tn-seq primer (first PCR)
**oTB536**	Mariner PCR1	GCCATCTATGTGTCTAGAGAC	Tn-seq primer (first PCR)

**oTB537**	Rnd2_Staph_IL	AATGATACGGCGACCACCGAGATCTACACTCTTTCGGGGACTTATCAGCCAACCTG	Tn-seq primer (second PCR)
**oTB538**	pLOW MCS seq f1	GACTTTATCTACAAGGTGTGGC	Colony PCR/sequencing primer for *pLOW* constructs
**oTB540**	pLOW MCS seq r1	TTCAGGCTGCGCAACTGTTG	Colony PCR/sequencing primer for *pLOW* constructs
**oTB545**	pGL485 fwd	taatgtATCGATaataatggtttcttagacg	Colony PCR for *pGL485*
**oTB546**	pGL485 rev	tattatGTCGACagtcggcattatctc	Colony PCR for *pGL485*
**oTB561**	pTP063d-01855 f	acaTAAGGAGGaGGTACCatgGATTGGATTTTACCAATTGCTGG	Subclone *facZ* into pTP63 vector; KpnI cut site
**oTB562**	pTP063d-01855 r	CCACCTGGAATTCttaTTTATCTACTCTAGAAGTATAGCTATGATTTGCATCAGTTGC	Subclone *facZ* into pTP63 vector; EcoRI cut site
**oTB563**	pTP063d-01855-mCh f	AGGAGGaGGTACCatgGATTGGATTTTACCAATTGCTGG	Subclone *facZ-mCherry* into pTP63 vector; KpnI cut site
**oTB564**	pTP063d-01855-mCh r	CACCTGGAATTCCttaGGATCCGCCAGCACCTTTG	Subclone *facZ-mCherry* into pTP63 vector; EcoRI cut site
**oTB566**	FtsZ-GFP KpnI F	GGAGGaGGTACCATGTTAGAATTTGAACAAGGATTTAATCATTTAGCG	Subclone *ftsZ-GFP* into pTP63 vector; KpnI cut site
**oTB568**	FtsZ-GFP EcoRI R	GAACGTCTTTCTTCTCTATTTCTAATGAAGC	Subclone *ftsZ-GFP* into pTP63 vector; EcoRI cut site
**oTB595**	GpsB cPCR F	GTTCTTCAAGCAGATGTTAGTTGATTTTATGG	Colony PCR for inactivation of *gpsB*
**oTB596**	GpsB cPCR R	GGACTTTCCTCTATATAATATAGCGATTACCC	Colony PCR for inactivation of *gpsB*
**oTB597**	GpsB Seq F	GTGATAAATTAAAAAATGTAGGAGGCGTCC	Colony PCR for inactivation of *gpsB*
**oTB598**	GpsB Seq R	CTTTCTTAGTTATCGCCTGACAATCTGGC	Colony PCR for inactivation of *gpsB*

**oTB599**	GpsB cPCR F	GGATAAAACAAACTATACTTGTGATATTGTG	Colony PCR for inactivation of *gpsB*
**oTB600**	GpsB cPCR R	CAATCATCTCAGACTGTGTGAGC	Colony PCR for inactivation of *gpsB*
**oTB689**	pLOW GFP Nterm ITA F	cctgcaggcatgcctgcagGTCGACacaTAAGGAGGaGGTACCATGAGTAAAGGAGAAGAACTTTTCACTGG	Generate GFP fragment for ITA of GFP-Pbp1
**oTB690**	pLOW GFP Nterm ITA R	CAGACCAGCCGGACCCTCGAGTTTGTATAGTTCATCCATGCCATGTGTAATCC	Generate GFP fragment for ITA of GFP-Pbp1
**oTB691**	Linker-PBP1 ITA F	CTCGAGGGTCCGGCTGGTCTGATGGCGAAGCAAAAAATTAAAATTAAAAAAAATAAAATAGGGGC	Generate Pbp1 fragment for ITA of GFP-Pbp1
**oTB692**	PBP1-pLOW ITA R	GAATTCgagctcgcccggGGATCCTTAGTCCGACTTATCCTTGTCAGTTTTACTGTCAG	Generate Pbp1 fragment for ITA of GFP-Pbp1
**oTB699**	GFP pLOW Cterm ITA F	ggtggtagtggtggtagtggtggtATGAGTAAAGGAGAAGAACTTTTCACTGG	Generate GFP fragment for ITA of FtsW-GFP
**oTB700**	GFP pLOW Cterm ITA R	gccagtGAATTCgagctcgcccggGGATCCTTATTTGTATAGTTCATCCATGCCATGTGTAATCCC	Generate GFP fragment for ITA of FtsW-GFP
**oTB701**	pLOW-ftsW ITA F	cctgcaggcatgcctgcagGTCGACacaTAAGGAGGaGGTACCatgAAGAATTTTAGAAGTATTTTACGGTATATTGGT AAAACC	Generate FtsW fragment for ITA of FtsW-GFP
**oTB702**	ftsW-linker ITA R	CTCATaccaccactaccaccactaccaccATTAAATTGTCTTCTTATATCAACTTTTTGTTGTTGTTTCTTTCG	Generate FtsW fragment for ITA of FtsW-GFP
**AGS59**	pTP63 backbone F	GAATTCCAGGTGGCACTTTTCG	Amplification of pTP63
**AGS60**	pTP63 backbone R	GGTACCATCATACTCTATCAATGA	Amplification of pTP63
**AGS61**	GpsB F	GAGTATGATGGTACCgtttctaatgaggtggaaaaaATGTCAGATGTTTCATTGAAAT	Generate GpsB fragment for ITA of GpsB-mNeon
**AGS62**	GpsB R	AGCTCCACCAGCGCTACCACCACCTTTACCAAATACAGCTTTTTCT	Generate GpsB fragment for ITA of GpsB-mNeon
**AGS63**	mNeonGreen F	AGCGCTGGTGGAGCTGTGAGTAAAGGTGAGGAGGA	Generate mNeon fragment for ITA of GpsB-mNeon

**AGS64**	mNeonGreen R	TGCCACCTGGAATTCTTActtgtcgtcatcgtctttgtagtcTTTATACAACTCATCCATTCCCAT	Generate mNeon fragment for ITA of GpsB-mNeon
**oTS079**	rbs_{rpoB}-GpsB F	GCAGGTCGACCATAATTTTTGAGGGGTGAATCTGTATGTCAGATGTTTCATTGAAATTATCA	Generate GpsB-FLAG insert
**oTS080**	GpsB-FLAG	CCCGGGGATCCTTACTTGTCGTCATCGTCTTTGTAG	Generate GpsB-FLAG insert
**AMo67**	gpsB_Wanner_KO_30H_U_o 67	AAGATCAAAGTTTCTAATGAGGTGGAAAAA CATAAAAACAACTCGTAGCTTATCAAAG	Generate fragment for amplifying KanR marker (for deleting *gpsB*)
**AMo68**	gpsB_Wanner_KO_30H_D_o 68	GTAAGACAGTTAAACTTTTGTATTTAGTAA CAATGACCTAAGAGGTGTGG	Generate fragment for amplifying KanR marker (for deleting *gpsB*)
**oRWB123**	GpsB SUMO Fwd	TGAACAGATTGGCGGCATGTCAG	Insert for pSUMO-FacZ 3x(127-146)
**oRWB124**	GpsB 75 SUMO Rev	catggatccttattacgtagcaacacgaaggcgaag	Insert for pSUMO-FacZ 3x(127-146)
**oRWB125**	Sumo-FacZ 127 Fwd	TgaacagattggcggcGAAATTGCAGATAAGTGGCAA	Insert for pSUMO-GpsB(1-75)
**oRWB126**	Sumo-FacZ 145 Rev	ccatggatccttattaTGCCTTGTAGTTTGCAGATCC	Insert for pSUMO-GpsB(1-75)

All oligos used in the course of this study are noted in this table.

References

1 Omasits, U., Ahrens, C. H., Müller, S. & Wollscheid, B. Protter: interactive protein feature visualization and integration with experimental proteomic data. *Bioinformatics* **30**, 884-886, doi:10.1093/bioinformatics/btt607 (2014).

2 Fey, P. D. *et al.* A Genetic Resource for Rapid and Comprehensive Phenotype Screening of Nonessential Staphylococcus aureus Genes. *mBio* **4**, e00537-00512, doi:doi:10.1128/mBio.00537-12 (2013).

3 Pang, T., Wang, X., Lim, H. C., Bernhardt, T. G. & Rudner, D. Z. The nucleoid occlusion factor Noc controls DNA replication initiation in Staphylococcus aureus. *PLOS Genetics* **13**, e1006908, doi:10.1371/journal.pgen.1006908 (2017).

4 Wang, H., Claveau, D., Vaillancourt, J. P., Roemer, T. & Meredith, T. C. High-frequency transposition for determining antibacterial mode of action. *Nature Chemical Biology* **7**, 720-729, doi:10.1038/nchembio.643 (2011).

5 Youngman, P. J., Perkins, J. B. & Losick, R. Genetic transposition and insertional mutagenesis in Bacillus subtilis with Streptococcus faecalis transposon Tn917. *Proc Natl Acad Sci U S A* **80**, 2305-2309, doi:10.1073/pnas.80.8.2305 (1983).

6 Ben-Yehuda, S. & Losick, R. Asymmetric cell division in B. subtilis involves a spiral-like intermediate of the cytokinetic protein FtsZ. *Cell* **109**, 257-266, doi:10.1016/s0092-8674(02)00698-0 (2002).

7 Roney, I. J. & Rudner, D. Z. Two broadly conserved families of polyprenyl-phosphate transporters. *Nature* **613**, 729-734, doi:10.1038/s41586-022-05587-z (2023).

8 Liew, A. T. F. *et al.* A simple plasmid-based system that allows rapid generation of tightly controlled gene expression in Staphylococcus aureus. *Microbiology* **157**, 666-676, doi:https://doi.org/10.1099/mic.0.045146-0 (2011).

9 Rodriguez, M. D., Paul, Z., Wood, C. E., Rice, K. C. & Triplett, E. W. Construction of Stable Fluorescent Reporter Plasmids for Use in Staphylococcus aureus. *Frontiers in Microbiology* **8**, doi:10.3389/fmicb.2017.02491 (2017).
